# The Effects of Dynamic Stability Training with Inertial Load of Water on Dynamic Balance and Pain in Middle-Aged Women with Chronic Low Back Pain: A Randomized Clinical Trial

**DOI:** 10.3390/jfmk11010014

**Published:** 2025-12-29

**Authors:** Ha Yeong An, Shuho Kang, Il Bong Park

**Affiliations:** Department of Sports Rehabilitation, Busan University of Foreign Studies, Busan 46234, Republic of Korea; 20235369@office.bufs.ac.kr (H.Y.A.); 20236204@bufs.ac.kr (S.K.)

**Keywords:** chronic low back pain, middle-aged women, inertial load of water training, dynamic balance, y-balance test

## Abstract

**Background:** Chronic low back pain (CLBP) is a common musculoskeletal disorder among middle-aged women, often leading to impaired dynamic balance and increased fear of movement. This study aimed to investigate the effects of dynamic stability training using the inertial load of water on balance ability and pain in middle-aged women with CLBP. **Methods:** Twenty-nine participants aged 40–65 years with CLBP were randomly assigned to an experimental or control group. The experimental group wore a water-filled aquavest, and the control group wore a weighted vest. Both groups performed 12 weeks of dynamic stability training twice per week. Outcome measures included the Y-Balance Test and Center of Pressure parameters, Visual Analogue Scale and Tampa Scale for Kinesiophobia. Data were analyzed using mixed-design two-way repeated-measures (between–within) analysis of variance to examine time, group, and interaction effects. **Results:** A significant group × time interaction effect was found in Y-Balance Test reach distances of the non-dominant leg, with the aquavest group showing greater improvements compared to the control group (*p* < 0.05). Center of Pressure analysis revealed decreased non-dominant leg Anterior–Posterior Root Mean Square in the aquavest group, indicating enhanced postural stability. Both groups showed decreased VAS and TSK. **Conclusions:** Dynamic stability training using inertial load of water effectively improved both quantitative and qualitative aspects of dynamic balance in middle-aged women with CLBP and can serve as a functional intervention for neuromuscular rehabilitation.

## 1. Introduction

Chronic low back pain (CLBP) is defined as low back pain that persists for more than 12 weeks without a clearly identifiable cause. It is one of the most prevalent musculoskeletal disorders among middle-aged women [1,2,3]. The higher prevalence observed in women is attributable to the combined effects of menopause-related declines in estrogen levels, pregnancy and childbirth, reductions in muscle mass and bone mineral density, and psychological factors [4,5,6,7]. CLBP often leads to difficulties in performing daily activities, impaired balance [8,9], and kinesiophobia, which results in avoidance of physical activity [10,11]. Such avoidance behaviors contribute to atrophy and weakening of the deep stabilizing muscles, thereby reducing spinal stability and increasing postural sway during standing [12,13]. Consequently, dynamic balance ability becomes further compromised [14]. These physiological and neuromuscular changes are closely associated with impairments in postural control and dynamic balance in middle-aged women with CLBP.

Balance is a complex sensorimotor function that integrates vestibular input, proprioceptive feedback from muscles and joints, and visual information to maintain posture and respond appropriately to external perturbations [15,16]. Dynamic balance, in particular, is essential for gait, directional changes, and the performance of other functional movements, and it is closely associated with fall prevention [17,18,19]. Individuals with CLBP often exhibit impaired postural control and reduced dynamic balance due to diminished proprioception and delayed activation of the deep stabilizing muscles [12,14]. Therefore, accurate and multidimensional assessment and improvement of dynamic balance are considered essential objectives in the rehabilitation of individuals with CLBP [12,20,21].

The Star Excursion Balance Test (SEBT) and the Y-Balance Test (YBT) are widely used methods for evaluating dynamic balance. The YBT, a simplified version of the SEBT, has been widely adopted in clinical and research settings due to its high reliability [22,23]. Using standardized equipment, the YBT measures an individual’s maximal reach distance, which serves as a quantitative outcome reflecting strength, flexibility, and neuromuscular control [21,24]. However, this measurement captures only how far the participant reaches and does not reflect the qualitative processes involved in reaching—such as postural control or balance strategies. In other words, identical reach distances may be produced through different postural control strategies. This limitation makes it difficult for conventional YBT scoring to distinguish among such strategic variations.

To address the limitations of dynamic balance tests that rely on a single outcome measure, recent studies have begun incorporating tools such as force plates and three-dimensional motion analysis systems to examine both the mechanical characteristics of movement and the accompanying postural control strategies [25,26,27]. Therefore, evaluating center of pressure (COP) variables as complementary qualitative outcomes, while retaining reach distance as the primary outcome measure of the YBT, can provide meaningful insights into inefficient postural control strategies employed by individuals with CLBP.

Core stabilization exercises [28] and unstable-surface training [29,30] have been widely used in the management of CLBP to enhance neuromuscular control and trunk stability. Unstable-surface exercises require continuous postural adjustments, promoting trunk muscle activation and sensorimotor integration, and have been shown to improve pain and functional outcomes in individuals with CLBP [29,30]. However, conventional unstable-surface training mainly depends on support surface instability, limiting the ability to actively modulate the direction and magnitude of external loads during movement [31]. To overcome these limitations, perturbation-based training approaches have recently gained attention as a novel strategy in individuals with CLBP [32,33]. One such training approach is dynamic stabilization training that utilizes the inertial load of water [34,35,36,37,38,39].

Previous studies have reported that training incorporating the inertial load of water enhances sensorimotor control, including proprioception, and induces neuromuscular adaptations essential for postural regulation in healthy adults as well as in middle-aged and older populations [35,36,37,38]. In addition, a study involving middle-aged women with degenerative knee arthritis confirmed that such training improved mediolateral postural stability and reduced pain, thereby enhancing balance function and promoting safer stair ambulation [39]. However, to date, no studies have directly investigated the effects of exercise using the inertial load of water in individuals with CLBP.

Therefore, the purpose of this study was to investigate the effects of dynamic stability training using the inertial load of water on dynamic balance and pain in middle-aged women with CLBP. Changes in dynamic balance were primarily assessed using reach distance in the YBT as the primary outcome measure, while COP variables were included as secondary outcomes to examine characteristics of postural stabilization strategies. Pain-related outcomes were additionally evaluated using the Visual Analogue Scale (VAS) and the Tampa Scale for Kinesiophobia (TSK) as secondary outcome measures.

The following hypotheses were proposed:

(1) The dynamic stability training group using the inertial load of water would demonstrate different patterns of change over time in anterior (AT), posteromedial (PM), and posterolateral (PL) reach distances of the YBT compared with the control group.

(2) The intervention would lead to improvements in COP range, velocity, and root mean square (RMS) during YBT performance, reflecting enhanced postural control strategies.

(3) Changes in YBT and COP variables in response to the intervention would differ between the dominant and non-dominant lower limbs.

## 2. Materials and Methods

### 2.1. Participants

A total of 30 middle-aged women with CLBP, aged between 40 and 65 years, were recruited for this study through local community postings and a university located in region B city. Before enrollment, all participants were informed about the study’s objectives and procedures, and written informed consent was obtained from each participant. This study was approved by the Institutional Review Board (IRB approval number: P01-202507-01-016) and has been registered with the Clinical Research Information Service (CRIS; registration number: KCT0011204).

Participants were randomly assigned to either the experimental group (AG, n = 14) or the control group (CG, n = 15). Randomization was conducted after the pre-assessment using a simple lottery method. Thirty slips labeled “A” or “B” were placed in an opaque, sealed box, and participants were assigned according to the slip they drew, ensuring random allocation.

**Figure 1:**
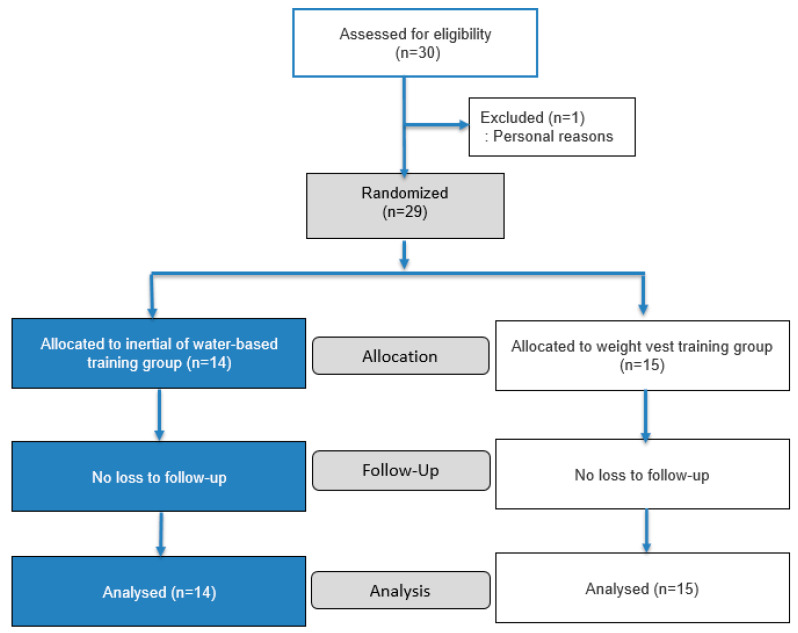
Flow diagram of the study participants.

All outcome assessments were conducted by the same investigator following standardized testing protocols. Due to the noticeable differences in appearance and perceived load characteristics between the intervention devices, participant blinding was not feasible. In addition, assessor blinding could not be achieved because the same investigator administered the intervention and conducted the assessments. To minimize potential bias, all quantitative outcomes, including YBT reach distances and COP variables, were collected using objective measurement systems. During the intervention period, one participant withdrew due to personal reasons; therefore, data from 29 participants were included in the final analysis (Figure 1).

The required sample size was calculated using the G*Power 3.1 software for a mixed design repeated-measures ANOVA (2 groups × 3 time points) focusing on interaction effects. Based on the previous study by Kang et al. [37], which examined balance-related outcomes such as postural sway following dynamic neuromuscular stabilization training, the effect size was set at f = 0.25. An alpha level of 0.05 and a statistical power (1 − β) of 0.80 were applied. The calculation indicated that a minimum of 24 participants, with 12 in each group, was required. Considering a 25% dropout rate, 30 participants were recruited to ensure adequate statistical power.

Inclusion criteria were as follows:

(1) Women aged 40–65 years diagnosed with CLBP lasting more than 3 months

(2) A score of 4 or higher on the VAS during pre-assessment

(3) The ability to voluntarily participate in the exercise program.

Exclusion criteria were as follows:

(1) History of abdominal, lumbar, or lower extremity surgery within the past year;

(2) Severe pain during walking or exercise that interfered with daily activities;

(3) Medical conditions considered inappropriate for participation by the investigator, including cardiopulmonary disease (e.g., heart failure, myocardial infarction, or chronic pulmonary disease) or current use of anxiolytics, antidepressants, or sedatives;

(4) Engagement in regular exercise at least three times per week during the past 6 months.

A homogeneity test revealed no significant differences between groups in baseline characteristics, including age (*p* = 0.474), height (*p* = 0.267), and weight (*p* = 0.602), or BMI (*p* = 0.280), indicating that the two groups were comparable at baseline. The participants’ general characteristics are summarized in Table 1.

### 2.2. Assessment of Dynamic Balance

The primary outcome measure of this study was the YBT, used to assess dynamic balance performance in individuals with CLBP. Secondary outcome measures included COP variables recorded during YBT performance, pain intensity assessed using the VAS, and the Korean version of TSK.

All dynamic balance assessment and data collections were performed by the same investigator to ensure consistency. Leg dominance was determined prior to testing based on the participants’ self-reported preferred kicking leg, with the majority identifying the right leg as dominant.

Participants wore short-sleeved shirts and tights to allow for accurate assessment of the lower limbs and performed the YBT after receiving sufficient instruction and practice. During the test, participants stood barefoot based on their leg preference on the central platform with hands on hips and the stance foot aligned along the central start line. The opposite leg was extended as far as possible in 3 directions—AT, PM, and PL.

To allow for familiarization, participants completed 3 practice trials for each leg, followed by a 2 min rest period. The main measurement consisted of 5 trials, and the average of 3 best trials was used for analysis. A trial was considered invalid and excluded if the participant (1) lifted the heel of the stance foot, (2) kicked the reach indicator, or (3) failed to return the reaching foot to the start position under control.

Reach distance was measured using the Y-Balance Test Kit, and the collected reach distance was normalized (%) by leg length, measured from the anterior superior iliac spine (ASIS) to the medial malleolus.

COP data during the YBT were collected using a force platform (AMTI-OR6 Force platforms, Watertown, MA, USA) integrated with the VICON Plug-in gait system (Vicon Nexus ver. 2.15, Oxford Metrics, Oxford, UK) at a sampling frequency of 100 Hz. Raw data were filtered using a fourth-order Butterworth low-pass filter with a cutoff frequency of 6 Hz.

**Figure 2:**
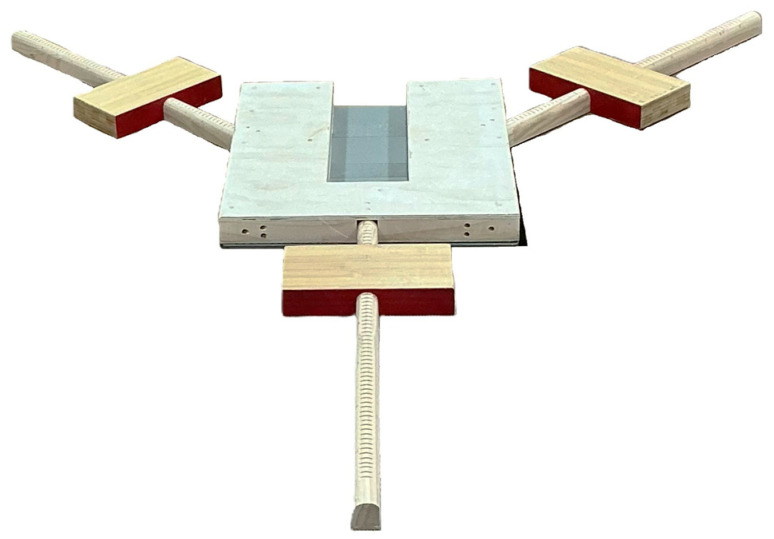
Set-up for measuring YBT & COP.

**Figure 3:**
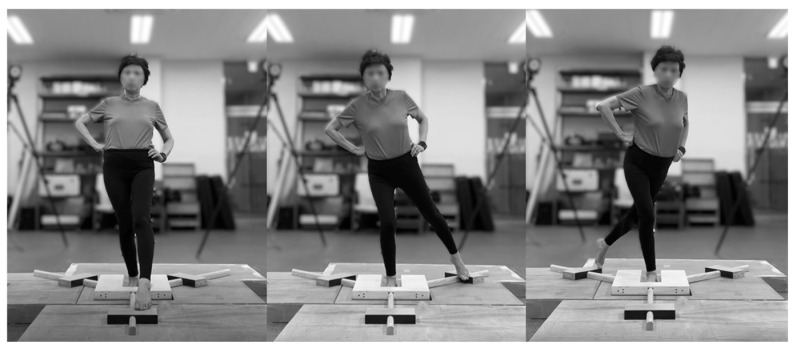
Y-Balance Test ((**left**): AT; (**center**): PM; and (**right**): PL).

The dynamic balancing test was performed on a custom-built wooden frame (width: 46.4 cm, length: 50.8 cm, height: 4.7 cm) securely mounted on the force plate to ensure stable installation of the YBT apparatus (Figure 2 and Figure 3). During the reach of the moving leg, the stance leg was used to analyze postural stability. COP variables—including mediolateral (ML) and anteroposterior (AP) range, velocity, and root mean square (RMS)—were selected to capture complementary aspects of dynamic postural control. Specifically, COP range reflects the overall excursion of the center of pressure, velocity represents the rate of postural adjustments, and RMS indicates the magnitude of COP variability during the task. These variables have been commonly used to characterize postural control strategies under dynamic and unstable conditions rather than quiet standing [40].

To account for inter-individual differences in task duration, COP velocity was normalized over time. COP data were analyzed over the entire reach phase, from initiation of the reaching movement to return to the starting position, to reflect continuous postural regulation during dynamic reaching rather than static stabilization at a single time point. All COP variables were analyzed separately for dominant and non-dominant legs.

### 2.3. Assessment of Pain

Pain intensity and movement-related fear were assessed using the VAS and the validated Korean version of the TSK, respectively.

The VAS is a 10-point scale ranging from 0, indicating no pain and the ability to perform daily activities without discomfort, to 10, indicating unbearable and persistent pain that completely interferes with daily functioning. Participants were asked to mark the point that best represented their current pain level.

The TSK consists of 17 items rated on a 4-point Likert scale ranging from 1 for ‘strongly disagree’ to 4 for ‘strongly agree’. The total score ranges from 17 to 68, with scores of 37 or higher indicating a high level of kinesiophobia.

### 2.4. Exercise Intervention

The dynamic stability training (DST) program was conducted twice per week for 12 weeks. Both the AG and the CG performed the same exercise protocol, which was adapted from the program proposed by Kang and Park [36] and modified for middle-aged women with chronic low back pain. All sessions were supervised by the principal investigator to ensure proper execution and safety.

**Figure 4:**
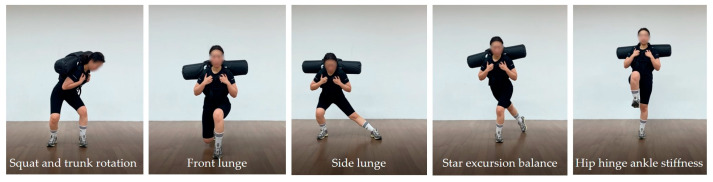
Dynamic stability training program performed for 12 weeks.

Each session consisted of a warm-up, a main exercise phase, and a cool-down, and lasted approximately 50 min. During the main exercise phase, participants performed progressive dynamic stability tasks including double-leg stance exercises, dynamic weight-shift transitions, and single-leg postural control exercises designed to enhance neuromuscular coordination and postural stability (Figure 4). The overall structure and detailed contents of the training program are presented in Table 2. To compare the effects of the intervention consistently, participants in both groups performed the same exercises at the same time.

**Figure 5:**
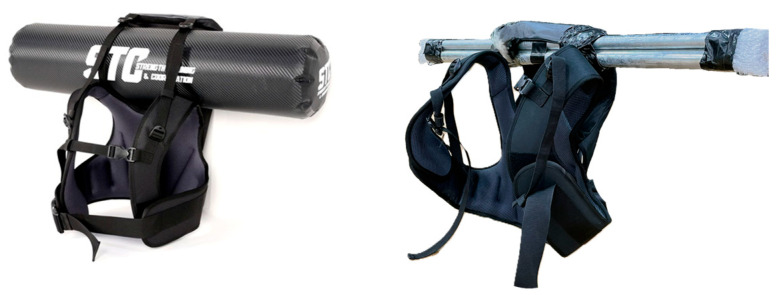
Structural differences between the aquavest (**Left**) and weighted vest (**Right**) used during the intervention.

The two groups differed only in the type of vest worn during exercise. Participants in the AG performed the exercises while wearing an aquavest that provided inertial water-based loads. The aquavest contained freely moving water, which generated continuous multidirectional load perturbations during movement. In contrast, participants in the CG performed the identical exercises while wearing a weighted vest containing fixed metal rods. This vest provided an equivalent total mass but produced a stable, unidirectional load without internal mass displacement (Figure 5).

For both groups, the vests were fitted using the same wearing configuration and adjusted to each participant’s body size, with the total load standardized at 3 kg. The fixed load was selected to ensure consistency between groups and to maintain clinical feasibility and safety for middle-aged women with chronic low back pain, while minimizing excessive mechanical stress. Exercise intensity was additionally regulated using the Rating of Perceived Exertion (RPE) scale [41], with all participants instructed to maintain a moderate intensity level (RPE 9–11). This combined approach allowed relative exercise intensity to be individually adjusted according to each participant’s physical capacity, despite the use of a standardized external load, thereby promoting comparable perceived exertion across participants.

### 2.5. Statistical Analysis

All statistical analyses were performed using IBM SPSS Statistics for Windows, version 25.0 (IBM Corp., Armonk, NY, USA). Descriptive statistics for all variables were calculated and are presented as mean ± standard deviation (SD). The independent *t*-test was used to verify the homogeneity of baseline demographic characteristics between the two groups. The Shapiro–Wilk test was conducted to assess the normality of the data distribution. To examine the effects of the intervention, a mixed-design two-way repeated measures ANOVA (group × time) was performed to compare outcome variables across groups and over time. When significant main or interaction effects were detected, Bonferroni-adjusted post hoc tests were applied for pairwise comparisons to identify specific within-groups differences across the time points. Although Bonferroni-adjusted post hoc tests were applied to control for multiple comparisons, the possibility of inflated Type I error cannot be completely excluded. The YBT reach distance was interpreted based on *p*-values, whereas secondary outcomes, including COP variables and pain measures, were interpreted using confidence intervals and effect sizes. Effect sizes (η^2^) were calculated and interpreted according to Cohen’s guidelines as small (0.01), medium (0.06), and large (0.14). The statistical significance level for all analyses was set at *p* < 0.05.

## 3. Results

The results related to dynamic balance measures YBT and COP and pain-related measures VAS and TSK are presented in Table 3, Table 4, Table 5, Table 6 and Table 7.

### 3.1. YBT Reach Distance by Direction

A significant time × group interaction was observed in the AT direction for both the non-dominant (*p* = 0.002, η^2^ = 0.398) and dominant legs (*p* = 0.031, η^2^ = 0.197), whereas significant interactions in the PM (*p* = 0.021, η^2^ = 0.501) and PL (*p* = 0.047, η^2^ = 0.377) directions were found only in the non-dominant leg.

A significant main effect of time was observed across all YBT directions for both legs in both groups. (all *p* < 0.01).

Post hoc analyses indicated that the AG showed significant improvements across all directions, whereas the CG exhibited minimal changes with limited statistical significance.

The post hoc power analysis for the AT reach distance of the dominant leg demonstrated a large time × group interaction effect (f = 0.50, achieved power > 0.99), indicating sufficient statistical power to detect the observed effect.

### 3.2. COP of Dynamic Balance

For the non-dominant leg, a significant time × group interaction was observed only for the AP RMS (η^2^ = 0.188). Post hoc analyses revealed a significant reduction in the AP RMS in the AG from week 0 to week 12 (95% CI [0.059, 0.744], η^2^ = 0.679) and from week 6 to week 12 (95% CI [0.183, 0.606], η^2^ = 0.882), whereas no significant changes were observed in the CG.

### 3.3. VAS and TSK

For VAS, a significant main effect of time was observed (Week 0 (95% CI [4.248, 5.466]) to Week 6 (95% CI [2.515, 4.485]) and Week 12 (95% CI [1.330, 1.956], η^2^ = 0. 641), indicating a reduction in pain levels over the intervention period, with no significant group or time × group interaction.

Similarly, TSK demonstrated a significant main effect of time (Week 0 (95% CI [39.782, 46.218]) to Week 6 (95% CI [38.298, 43.702]) and Week 12 (95% CI [33.975, 40.454], η^2^ = 0.235), indicating an overall decrease in fear of movement, with no significant group or interaction effects.

## 4. Discussion

This study investigated the effects of 12 weeks of dynamic stabilization training using the inertial load of water on middle-aged women with CLBP, comparing the AG with the CG. The YBT was designated as the primary outcome to assess changes in dynamic balance, while COP measures were included as secondary outcomes to examine alterations in postural stabilization strategies during a functional reaching task. The results demonstrated that the AG showed significantly greater improvement in YBT reach distance on the non-dominant leg compared with the CG. In addition, between-group differences were observed in the AP RMS of the COP on the non-dominant leg suggesting changes in postural regulation under perturbation-based conditions.

In this study, the YBT was selected to assess dynamic balance in individuals with CLBP. The YBT evaluates flexibility, neuromuscular control, proprioception, and strength, making it a widely used clinical tool for assessing postural control in this population [12,21]. Furthermore, the YBT-LQ has consistently demonstrated high reliability across multiple reliability studies and systematic reviews, and its validity and reliability have been confirmed in CLBP populations, supporting its appropriateness for the present study [23]. The test protocol is standardized, including leg length normalization, left-right asymmetry analysis, and composite score calculation—enhances both clinical applicability and comparability across studies. Additionally, the YBT is time-efficient, does not require expensive equipment, and allows repeated measurements, making it suitable for intervention studies involving multiple participants [42]. Accordingly, YBT performance was used as the primary indicator of dynamic balance in this study.

The AG showed a significant improvement in anterior reach distance compared with the CG. The water inside the aqua vest exhibits nonlinear and time-delayed responses due to its unique fluid properties, in contrast with stable loads [34,35]. These characteristics generate continuous and unpredictable perturbations to the wearer’s trunk and lower limbs [35,36,39,43]. Such an unstable environment requires sustained postural regulation and may promote adaptations in postural control strategies beyond those elicited by conventional resistance training [37,38]. During this process, the central nervous system may increasingly rely on anticipatory muscle activation and intermuscular coordination, reflecting enhanced integration of feedforward and feedback control [37,38,39,44]. Consequently, the observed between-group differences may be interpreted as reflecting adaptive responses to velocity- and acceleration-dependent resistance induced by the inertial load of water, as well as direction-specific perturbations generated by fluid motion. These characteristics suggest that movement execution and balance control strategies in the AG may have differed from those elicited by the more stable resistance conditions applied in the CG [38,39,45].

The differences in load characteristics were reflected in the significant Group × Time interaction observed for AT reach distance in the YBT. Forward reaching requires coordinated activation of the ankle and trunk musculature to shift the center of mass anteriorly while maintaining anticipatory postural stabilization [12,46]. Such trunk–lower limb coordination is often impaired in individuals with CLBP [21,47]. Moreover, middle-aged adults commonly exhibit age-related declines in sensory integration and delayed reaction times, which have been associated with reduced reach performance during dynamic balance tasks [48]. In contrast to weighted vests, which provide a relatively constant gravitational load with minimal directional variation [49,50], the inertial load of water produces unstable and movement-dependent perturbations that may impose greater demands on anticipatory postural control. These differences in physical load characteristics may lead to distinct patterns of motor learning and neuromuscular adaptation, which may help explain the Group × Time interaction observed for AT reach distance in the YBT [35,36].

The greater improvement in AT reach distance on the non-dominant leg observed in in the AG may be partially attributable to the unilateral weight-bearing exercises included in the intervention program, such as lunges. Lunge exercises require weight-bearing, trunk stabilization and coordinated control of the ankle, knee, and hip joints under load-bearing conditions [51,52]. When performed with the inertial load of water, these exercises repeatedly expose participants to external perturbations, which may have promoted feedback-based control and refinement of previously inefficient postural strategies in the non-dominant leg [38,39]. Previous studies have reported that the non-dominant leg typically exhibits lower sensorimotor integration and greater reliance on feedback-based control compared with the dominant leg [53,54,55]. Considering these characteristics, repeated exposure to perturbation-based lunge training using the inertial load of water appears to have contributed, at least in part, to the observed improvements in anterior reach performance on the non-dominant side.

Individuals with CLBP have been reported to rely excessively on ankle-dominant postural strategies while demonstrating reduced engagement of the hip strategy during balance tasks [56,57,58]. The DST applied in this study included exercises such as squats and lunges, which require hip mobility and control. The complex perturbations generated by the inertial load of water may have further facilitated proprioceptive re-education and postural adaptation. These training components may have supported the reorganization of movement strategies, promoting more effective coordination between hip and ankle strategies during functional tasks. Nevertheless, these interpretations should be considered indirect, as no direct neuromuscular measurements were obtained.

Previous studies have reported increased COP displacement in the AP direction during quiet standing in individuals with CLBP, reflecting impaired postural control and inefficient muscle coordination [59,60]. In the present study, AP RMS of the COP on the non-dominant leg demonstrated between-group differences, with post hoc analysis indicating a tendency for reduction only in the AG. This finding contrasts with previously reported CLBP patterns and suggests that exposure to perturbations induced by the inertial load of water may have promoted adaptive postural regulation. However, the interpretation of COP behavior during dynamic balance tasks is inherently complex, as COP variability reflects not only postural stability but also task-specific movement strategies and adaptive responses to external perturbations. Therefore, the observed reduction in non-dominant leg AP RMS in the aqua vest group should be interpreted as an indirect indicator of improved postural regulation rather than a direct measure of neuromuscular efficiency [36,38,59,61].

Both groups showed similar reductions in VAS and TSK scores over the 12-week intervention. Self-reported pain measures and performance- or biomechanical-based measures reflect different aspects of function, and their responsiveness may vary across participants [62,63]. This may explain the lack of a significant time × group interaction. Previous studies also suggest that pain scales in chronic low back pain reflect not only pain experience but complex musculoskeletal factors [62,63]. Thus, functional improvements following interventions such as DST may arise through multiple pathways, including neuromuscular retraining as well as psychological and neurophysiological mechanisms [64,65,66,67]. In contrast, YBT and COP primarily reflect mechanical adaptations. Therefore, the absence of a time × group interaction in VAS and TSK scores should not be interpreted as a lack of treatment effect but as evidence of the multifactorial nature of recovery.

This study has the following limitations:

1. Although improvements in dynamic balance may have occurred, the lack of direct neurophysiological measures limits the interpretation of the results.
2. The study included only middle-aged women with CLBP and did not classify subtypes or pain patterns, which limits generalizability to the broader CLBP population.
3. Despite methodological controls, the potential influence of novelty effects or participant expectations on the outcomes cannot be entirely ruled out.
4. The study focused on comparing the effects of specific interventions, which limits the generalizability of the findings.

In this study, analyses focused on functional performance and COP measures during dynamic balance tasks; however, mechanisms such as neuromuscular control, proprioceptive adaptation, and strategic postural adjustments could not be directly examined. Future studies should incorporate direct measurements, including electromyography, kinematics, and proprioceptive assessments, and refine postural control evaluation by normalizing reach distance with anthropometric parameters such as foot length.

Clinically, the present intervention may be applicable to middle-aged women with CLBP who are capable of performing moderate-intensity exercise. However, because the study did not classify participants by pain type and was limited in terms of sex and age, caution is required when generalizing the findings to the broader CLBP population. In addition, the 12-week intervention period did not allow for the assessment of long-term effects, leaving uncertainty regarding whether the duration was sufficient.

Differences in load and sensory experience between the Aquavest and Weighted vest may impose some limitations on interpreting the intervention effects. In addition, the potential influence of psychological factors, such as participants’ expectations on subjective pain measures like VAS and TSK, could not be entirely ruled out. Therefore, it is necessary to investigate the interaction between sensory experience differences and participant expectations on subjective pain outcomes.

Due to the relatively small sample size, the focus on middle-aged women, and the lack of long-term follow-up, caution is required when generalizing the findings to the broader CLBP population. This study provides preliminary data directly comparing two intervention strategies, and future research should include comparisons with a non-exercising control group as well as validation with larger samples.

The findings of this study partially support the initial hypotheses. Participants in the dynamic stability training group using the inertial load of water showed significant improvements in anterior reach distance of the non-dominant leg, while changes in posteromedial and posterolateral reach distances were not significant. COP variables indicated task-specific postural adaptations, although improvements were limited and should be interpreted cautiously. Differential responses between the dominant and non-dominant lower limbs were observed, indicating that perturbation-based dynamic stability training with the inertial load of water selectively enhances aspects of dynamic balance and postural control in middle-aged women with chronic low back pain.

## 5. Conclusions

This 12-week study suggests that DST incorporating the inertial load of water may contribute to improvements in selected aspects of dynamic balance in middle-aged women with CLBP. Specifically, greater improvements in anterior reach distance of the non-dominant leg were observed in the AG compared with the CG. Both groups demonstrated reductions in pain over time, indicating that participation in structured exercise itself may play an important role in pain-related outcomes. Overall, these findings suggest that the use of the inertial load of water may be considered an additional option for targeting task-specific dynamic postural control within exercise programs for individuals with CLBP.

## Figures and Tables

**Table 1:** Characteristics of study participants (n = 29).

	AG (n =14)	CG (n = 15)
Age (years)	57.85 ± 5.30	59.20 ± 4.66
Weight (kg)	59.31 ± 12.73	54.67 ± 9.13
Height (cm)	159.04 ± 4.16	158.19 ± 4.48
BMI (kg/m^2)^	23.34 ± 4.31	21.79 ± 3.21

Values are presented as means ± standard deviations. AG, aquavest group; CG, control group.

**Table 2:** Dynamic stability training program.

Training	0~6 Weeks	7~12 Weeks	Time
Intensity	9~11 RPE with weight (3 kg) 1~2 sets	9~11 RPE with weight (3 kg) 3 sets	
Warm-up	Spine Stretch & Hip Joint, Ankle Mobility	Spine Stretch & Hip Joint, Ankle Mobility	10 min
DST exercise	1. Two-leg support (Squat) 2. Step movement (front lunge/side lunge/star excursion balance) 3. Single-leg support (one-leg balance)	1. Two-leg support (Squat and trunk rotation) 2. Step movement (front lunge/side lunge/star excursion balance) 3. Single-leg support (one-leg balance/hip hinge ankle stiffness)	30 min
Cool down	Cat Stretch & Bear Position Hip Flexors & Extensors & Rotators Stretch	Cat Stretch & Bear Position Hip Flexors & Extensors & Rotators Stretch	10 min

**Table 3:** YBT for non-dominant leg reach distance by direction.

Variable	Group	0 Weeks	6 Weeks	12 Weeks	Source	*p*	η^2^	Post Hoc
AT (%)	AG	62.18 ± 5.30	66.26 ± 6.00	69.41 ± 5.46	Time	<0.001	0.398	0–6 w: 0.011 0–12 w: <0.001 6–12 w: 0.017
	CG	62.38 ± 8.17	66.71 ± 7.65	64.33 ± 6.19	Group × Time	0.002	0.200	0–6 w: 0.005 0–12 w: 0.423 6–12 w: 0.080
PM (%)	AG	91.67 ± 11.18	99.28 ± 6.62	106.25 ± 7.46	Time	<0.001	0.501	0–6 w: 0.004 0–12 w: <0.001 6–12 w: 0.001
	CG	90.51 ± 9.55	92.39 ± 9.35	97.05 ± 11.05	Group × Time	0.021	0.133	0–6 w: 1.000 0–12 w: 0.018 6–12 w: 0.031
PL (%)	AG	89.39 ± 13.27	97.11 ± 6.21	102.66 ± 8.77	Time	<0.001	0.377	0–6 w: 0.015 0–12 w: <0.001 6–12 w: 0.001
	CG	87.05 ± 10.21	91.41 ± 9.68	91.66 ± 9.98	Group × Time	0.047	0.122	0–6 w: 0.256 0–12 w: 0.279 6–12 w: 1.000

Note: Data are presented as mean ± standard deviation. AT: anterior; PM: posteromedial; PL: posterolateral; AG: aquavest group; CG: control group; and *p*-values are based on mixed-design two-way ANOVA. Post hoc comparisons were adjusted using the Bonferroni correction. η^2^: partial eta squared (small = 0.01, medium = 0.06, large ≥ 0.14).

**Table 4:** YBT for dominant leg reach distance by direction.

Variable	Group	0 Weeks	6 Weeks	12 Weeks	Source	*p*	η^2^	Post Hoc
AT (%)	AG	63.39 ± 5.66	65.64 ± 5.36	69.02 ± 5.22	Time	0.006	0.197	0–6 w: 0.537 0–12 w: 0.001 6–12 w: 0.006
	CG	62.25 ± 7.43	64.75 ± 7.76	63.29 ± 6.82	Group × Time	0.031	0.133	0–6 w: 0.369 0–12 w: 1.000 6–12 w: 0.416
PM (%)	AG	91.40 ± 10.28	99.49 ± 7.49	102.27 ± 6.49	Time	<0.001	0.349	0–6 w: 0.013 0–12 w: <0.001 6–12 w: 0.356
	CG	90.33 ± 8.84	95.95 ± 10.91	94.89 ± 9.61	Group × Time	0.138	0.071	0–6 w: 0.100 0–12 w: 0.170 6–12 w: 1.000
PL (%)	AG	89.46 ± 10.15	96.31 ± 9.49	100.97 ± 9.99	Time	<0.001	0.358	0–6 w: 0.023 0–12 w: <0.001 6–12 w: 0.053
	CG	87.43 ± 10.93	92.00 ± 10.38	92.51 ± 8.53	Group × Time	0.118	0.076	0–6 w: 0.170 0–12 w: 0.114 6–12 w: 1.000

Note: Data are presented as mean ± standard deviation. AT: anterior; PM: posteromedial; PL: posterolateral; AG: aquavest group; CG: control group; and *p*-values are based on mixed-design two-way ANOVA. Post hoc comparisons were adjusted using the Bonferroni correction. η^2^: partial eta squared (small = 0.01, medium = 0.06, large ≥ 0.14).

**Table 5:** COP for non-dominant leg dynamic balance.

Variable	Group	0 Weeks	6 Weeks	12 Weeks	Source	*p*	η*^2^*	Post Hoc
AP range (mm)	AG	10.76 ± 3.14	10.44 ± 1.75	9.49 ± 1.86	Time	0.394	0.032	0–6 w: 1.000 0–12 w: 0.212 6–12 w: 0.115
	CG	9.45 ± 2.67	10.11 ± 1.80	9.99 ± 1.88	Group × Time	0.104	0.084	0–6 w: 0.826 0–12 w: 1.000 6–12 w: 1.000
ML range (mm)	AG	4.76 ± 2.15	7.46 ± 2.08	9.92 ± 3.22	Time	<0.001	0.485	0–6 w: 0.023 0–12 w: <0.001 6–12 w: 0.025
	CG	5.67 ± 2.24	7.85 ± 3.84	9.47 ± 3.36	Group × Time	0.554	0.022	0–6 w: 0.070 0–12 w: 0.001 6–12 w: 0.189
AP velocity (mm/s)	AG	16.40 ± 4.28	16.17 ± 2.31	13.74 ± 2.19	Time	0.002	0.272	0–6 w: 1.000 0–12 w: 0.069 6–12 w: <0.001
	CG	18.16 ± 4.13	15.93 ± 2.66	15.14 ± 3.41	Group × Time	0.251	0.050	0–6 w: 0.099 0–12 w: 0.026 6–12 w: 0.296
ML velocity (mm/s)	AG	12.54 ± 2.51	12.54 ± 1.84	11.76 ± 2.00	Time	0.047	0.115	0–6 w: 1.000 0–12 w: 0.875 6–12 w: 0.318
	CG	14.05 ± 2.78	12.76 ± 2.05	12.48 ± 2.50	Group × Time	0.344	0.038	0–6 w: 0.187 0–12 w: 0.108 6–12 w: 1.000
AP RMS (mm)	AG	2.40 ± 0.72	2.39 ± 0.47	1.99 ± 0.43	Time	0.034	0.118	0–6 w: 1.000 0–12 w: 0.018 6–12 w: <0.001
	CG	2.00 ± 0.48	2.17 ± 0.39	2.15 ± 0.39	Group × Time	0.007	0.188	0–6 w: 0.435 0–12 w: 0.824 6–12 w: 1.000
ML RMS (mm)	AG	1.05 ± 0.24	1.49 ± 0.35	2.05 ± 0.74	Time	<0.001	0.501	0–6 w: 0.065 0–12 w: <0.001 6–12 w: 0.008
	CG	1.18 ± 0.31	1.69 ± 0.88	2.00 ± 0.72	Group × Time	0.592	0.019	0–6 w: 0.024 0–12 w: <0.001 6–12 w: 0.190

Note: Data are presented as mean ± standard deviation (SD). AP: anterior–posterior; ML: medial–lateral; AG: aquavest group; CG: control group; and *p*-values are based on mixed-design two-way ANOVA. Post hoc comparisons were adjusted using the Bonferroni correction. η^2^: partial eta squared (small = 0.01, medium = 0.06, large ≥ 0.14).

**Table 6:** COP for dominant leg dynamic balance.

Variable	Group	0 Weeks	6 Weeks	12 Weeks	Source	*p*	η*^2^*	Post Hoc
AP range (mm)	AG	9.89 ± 2.42	10.08 ± 1.85	8.73 ± 1.61	Time	0.004	0.185	0–6 w: 1.000 0–12 w: 0.044 6–12 w: 0.006
	CG	9.50 ± 2.19	10.07 ± 2.31	9.42 ± 1.80	Group × Time	0.172	0.063	0–6 w: 0.473 0–12 w: 1.000 6–12 w: 0.303
ML range (mm)	AG	4.82 ± 2.06	6.98 ± 1.60	8.01 ± 2.13	Time	<0.001	0.469	0–6 w: 0.007 0–12 w: <0.001 6–12 w: 0.215
	CG	5.33 ± 1.68	6.29 ± 2.56	8.24 ± 2.99	Group × Time	0.364	0.037	0–6 w: 0.399 0–12 w: 0.001 6–12 w: 0.003
AP velocity (mm/s)	AG	15.91 ± 4.92	14.84 ± 3.17	13.46 ± 2.17	Time	0.010	0.182	0–6 w: 0.678 0–12 w: 0.024 6–12 w: 0.022
	CG	16.34 ± 2.82	15.91 ± 2.44	15.19 ± 3.02	Group × Time	0.436	0.027	0–6 w: 1.000 0–12 w: 0.517 6–12 w: 0.385
ML velocity (mm/s)	AG	12.33 ± 3.34	12.12 ± 2.49	11.48 ± 1.80	Time	0.320	0.041	0–6 w: 1.000 0–12 w: 0.639 6–12 w: 0.635
	CG	13.06 ± 2.96	12.54 ± 2.09	12.53 ± 2.60	Group × Time	0.744	0.009	0–6 w: 1.000 0–12 w: 1.000 6–12 w: 1.000
AP RMS (mm)	AG	2.10 ± 0.41	2.10 ± 0.45	2.04 ± 0.44	Time	0.495	0.026	0–6 w: 1.000 0–12 w: 1.000 6–12 w: 1.000
	CG	2.12 ± 0.51	2.19 ± 0.52	2.12 ± 0.40	Group × Time	0.783	0.009	0–6 w: 1.000 0–12 w: 1.000 6–12 w: 0.785
ML RMS (mm)	AG	1.04 ± 0.36	1.41 ± 0.34	1.60 ± 0.44	Time	<0.001	0.409	0–6 w: 0.029 0–12 w: 0.004 6–12 w: 0.257
	CG	1.12 ± 0.20	1.30 ± 0.56	1.69 ± 0.68	Group × Time	0.472	0.027	0–6 w: 0.513 0–12 w: 0.002 6–12 w: 0.002

Note: Data are presented as mean ± standard deviation (SD). AP: anterior–posterior; ML: medial–lateral; AG: aquavest group; CG: control group; and *p*-values are based on mixed-design two-way ANOVA. Post hoc comparisons were adjusted using the Bonferroni correction. η^2^: partial eta squared (small = 0.01, medium = 0.06, large ≥ 0.14).

**Table 7:** VAS and TSK.

Variable	Group	0 Weeks	6 Weeks	12 Weeks	Source	*p*	η*^2^*	Post Hoc
VAS	AG	4.86 ± 1.23	3.50 ± 2.28	1.64 ± 0.63	Time	<0.001	0.641	0–6 w: 0.031 0–12 w: <0.001 6–12 w: 0.004
	CG	4.60 ± 0.99	2.60 ± 1.18	1.60 ± 0.51	Group × Time	0.377	0.035	0–6 w: 0.001 0–12 w: <0.001 6–12 w: 0.162
TSK	AG	43.00 ± 5.38	41.00 ± 5.10	37.21 ± 4.34	Time	0.001	0.235	0–6 w: 0.347 0–12 w:0.009 6–12 w: 0.058
	CG	37.47 ± 6.29	37.47 ± 4.76	35.00 ± 7.06	Group × Time	0.295	0.044	0–6 w: 1.000 0–12 w: 0.477 6–12 w: 0.317

Note: Data are presented as mean ± standard deviation (SD). VAS: visual analogue scale; TSK: Tampa scale of kinesiophobia; AG: aquavest group; CG: control group; and *p*-values are based on mixed-design two-way ANOVA. Post hoc comparisons were adjusted using the Bonferroni correction. η^2^: partial eta squared (small = 0.01, medium = 0.06, large ≥ 0.14).

## Data Availability

The data used in this study are available upon reasonable request and will be deposited in a public repository upon publication.
